# Putative Effector Genes Reveal Hidden Genetic Diversity Beyond Conventional Typing Markers in Flavescence Dorée Phytoplasmas

**DOI:** 10.3390/pathogens15080850

**Published:** 2026-08-14

**Authors:** Camilla Barbieri, Sara Torcoli, Flavio Serina, Martino Salvetti, Ivo Ercole Rigamonti, Nicola Mori, Piero Attilio Bianco, Fabio Quaglino

**Affiliations:** 1 Department of Agricultural and Environmental Sciences-Production, Landscape, Agroenergy, University of Milan, via Celoria 2, 20133 Milan, Italy; camilla.barbieri@unimi.it (C.B.); piero.bianco@unimi.it (P.A.B.); 2 Franciacorta Consortium, via Verdi 53, 25030 Erbusco, Italy; s.torcoli@franciacorta.wine (S.T.); f.serina@franciacorta.wine (F.S.); 3 Fondazione Fojanini di Studi Superiori, Via Valeriana 32, 23100 Sondrio, Italy; msalvetti@fondazionefojanini.it; 4 Department of Food, Environmental and Nutritional Sciences, University of Milan, Via Celoria 2, 20133 Milan, Italy; ivo.rigamonti@unimi.it; 5 Department of Biotechnology, University of Verona, Via della Pieve 70, 37020 San Floriano, Italy; nicola.mori@univr.it

**Keywords:** grapevine yellows, phylogeny, molecular markers

## Abstract

Flavescence dorée (FD) is a severe grapevine disease associated with FD phytoplasmas (FDp), mainly transmitted by the insect vector *Scaphoideus titanus* and occurring also in symptomless reservoir plants. This study assessed the genetic variability of putative effector-coding genes (*hp*) in FDp and FDp-related strains from Northern Italy, assigned to epidemic (M54, M38, M50, M51) and non-epidemic (M39, M43, M78) *map* genotypes. Based on the reference genome of FDp strain CH, 20 *hp* loci were selected and analyzed through a multi-locus sequence typing approach. Sixteen loci were amplified in at least one genotype, while four loci failed to amplify under the conditions used. Sequence comparisons showed complete conservation of all amplified *hp* genes within M54 strains, whereas other genotypes displayed different divergence levels, with the greatest heterogeneity observed in the M50 genotype, including also intra-genotype variation. Several loci exhibited substantial strain-level variability, suggesting locus- and strain-dependent diversification. However, this variability was not consistently associated with biological or epidemiological traits of FDp strains, such as host plant or epidemic status, and its functional relevance therefore remains unresolved. Overall, these results provide a first characterization of sequence diversity in candidate virulence-related loci across FDp genetic backgrounds, revealing additional genetic diversity beyond conventional FDp typing markers and supporting further functional investigation of their possible role in host or vector interactions.

## 1. Introduction

Flavescence dorée (FD) is an epidemic grapevine disease associated with phytoplasmas, wall-less and phloem-restricted bacteria belonging to the class Mollicutes. FD belongs to the Grapevine Yellows (GY) complex, a group of diseases caused by genetically distinct phytoplasmas but characterized by similar symptoms, including leaf yellowing or reddening, downward curling, incomplete lignification of canes, and grape desiccation [1]. First reported in France in the 1950s [2], FD has rapidly spread across several European countries, where it represents a major threat to viticulture due to its strong impact on yield and the lack of curative treatments [3,4]. As a quarantine disease, its management relies on mandatory control measures, including the removal of infected plants and insecticide treatments targeting the main vector *Scaphoideus titanus* [5].

FD-associated phytoplasmas (FDp) belong to the ribosomal group 16SrV, a taxonomic group that also includes phytoplasmas associated with other woody hosts, such as alder (Alder yellows–Alder yellows phytoplasma, AldYp), elm (Elm yellows–‘*Ca*. P. ulmi’), *Rubus* spp. (Rubus stunt–‘*Ca*. P rubi’), and jujube (jujube witches’ broom–‘*Ca*. P. ziziphi’). Within this group, FDp have traditionally been classified into the subgroups 16SrV-C and 16SrV-D based on *16S rRNA* gene sequence analysis [6,7,8,9]. Although the *16S rRNA* gene is widely used for phytoplasma identification and taxonomic classification [10], its high sequence conservation provides limited resolution among closely related 16SrV strains and does not fully capture their genetic and ecological diversity [9,11,12].

To refine strain discrimination, additional molecular markers have been developed. Among these, the *map* gene, encoding methionine aminopeptidase, has been widely used for FDp typing and allowed the definition of a large number of genotypes divided into three clusters (Map-FD1, Map-FD2, and Map-FD3) differing in geographical distribution, host association, and epidemiological relevance [11,13,14,15]. Further resolution has been provided by markers such as *vmpA*, which discriminates strains according to their involvement in the grapevine-*S. titanus* epidemic cycle, and by *dnaK*, *malG*, and other recently identified molecular markers, which resolve FDp diversity at different epidemiological scales [14,16,17]. In contrast, the ribosomal 16SrV-C/16SrV-D classification has limited discriminatory power with respect to the epidemiological behaviour of FD-associated phytoplasmas. Strains from both subgroups have been detected in symptomatic *Vitis vinifera*, whereas their major biological differences concern the host–vector cycles in which they circulate. The 16SrV-D lineage and a subset of 16SrV-C genotypes are compatible with *S. titanus* and participate in the epidemic grapevine-to-grapevine transmission cycle (FDp *sensu stricto*), while other 16SrV-C genotypes circulate predominantly in alternative plant–vector systems and are not transmissible by *S. titanus* (FDp-related strains) [14]. This distinction is particularly relevant from an epidemiological perspective, as increasing evidence has demonstrated that FD epidemiology is not restricted to the classical grapevine-*S. titanus* pathosystem. Indeed, FDp and FDp-related strains have been detected in several wild plant species, generally symptomless, including *Alnus glutinosa*, *Clematis vitalba*, *Ailanthus altissima*, *Corylus avellana* and other woody or herbaceous hosts [11,18,19,20,21,22]. In addition, alternative insect vectors such as *Dictyophara europaea*, *Orientus ishidae*, *Allygus* spp., *Oncopsis alni*, *Lamprotettix nitidulus*, and the newly described species *Bamboosella dimorpha* [23] have been connected to the transmission of 16SrV phytoplasmas to grapevine [14,20,24,25,26,27,28]. These findings support a complex ecological scenario occurring at the landscape level in which FDp presence extends beyond cultivated vineyards.

The ability of phytoplasmas to colonize different plant hosts and insect vectors relies on highly specialized molecular interactions, which are largely mediated by effector proteins and membrane-associated proteins [29]. Phytoplasma effectors are small secreted proteins that can modulate host physiology, suppress plant defence responses, alter plant development, and influence vector behaviour [30]. They are typically secreted via the conserved Sec-dependent secretion system, which recognizes N-terminal signal peptides and mediates protein translocation across the phytoplasma membrane into the host environment [31,32]. In addition to secreted effectors, membrane-associated proteins also play a key role in phytoplasma-host interaction and pathogenicity, as the absence of a cell wall leaves phytoplasma membrane proteins directly exposed at the interface with plant and insect host cells. Among these, immunodominant membrane proteins (IDPs), including Imp and VmpA/B, have been identified as conserved group features in 16SrV phytoplasmas [33] and linked to vector specificity, adhesion, colonization, and transmission efficiency [34,35,36,37,38].

Despite the recognized relevance of secreted effectors and membrane-associated proteins in phytoplasma biology, the molecular determinants underlying FDp pathogenicity and host/vector interactions remain poorly understood. This gap is partly due to the lack of axenic cultivation systems, which constrain functional studies. Nevertheless, the recent availability of the complete genome sequence of the Swiss FDp strain CH [39] has provided a framework to explore candidate virulence-related genes. Genome annotation identified several hypothetical proteins as putative effectors, based on predicted functional motifs such as signal peptides and transmembrane domains.

In this context, the present study aimed to investigate the genetic variability of putative effector-coding genes in FDp and FDp-related strains detected in northern Italy. A multilocus sequence typing (MLST) approach was applied to a panel of strains representing different *map* genotypes, including isolates from grapevine and from asymptomatic wild host plants. This approach was used to determine whether candidate virulence-related loci show conserved or divergent patterns across FDp and FDp-related genetic backgrounds, particularly between the homogeneous 16SrV-D/M54 genotype and the more diverse 16SrV-C strains.

## 2. Materials and Methods

### 2.1. Plant Sample Selection

The present study was performed on DNA extracts obtained from grapevines and additional woody plants infected by FDp and FDp-related strains previously collected and characterized in two viticultural areas of northern Italy, Valtellina and Franciacorta [20,40] (Table 1). In the original studies, FDp detection and strain characterization were performed through *map* gene sequence analysis according to the classification proposed by Malembic-Maher et al. [14]. In detail, DNA extracts were selected based on their *map* genotype to obtain a representative panel of FDp and FDp-related strains from distinct geographic and ecological contexts. The selected panel included epidemic genotypes M54, M51, M50, and M38 (FDp genotypes), as well as non-epidemic genotypes M39, M43, and M78 (FDp-related genotypes). Among the available grapevine samples, five *Vitis vinifera* DNA extracts per area were selected, all belonging to the epidemic M54 genotype. Additional DNA extracts from FDp-infected spontaneous woody plants were selected to include different alternative host species and different *map* genotypes detected in the two areas (Table 1). The resulting *map* genotype information was used as the reference framework for the subsequent comparative analysis of candidate virulence-related loci.

### 2.2. Primer Design for MLST Analysis of Putative Effectors

To analyze the genetic diversity of putative effector and virulence-related genes across FDp and FDp-related strains assigned to different *map* genotypes, a multilocus sequence typing (MLST) approach was developed. Target gene selection was based on the complete genome sequence of the FDp reference strain CH (GenBank accession no. CP097583), published by Debonneville et al. [39], in which 21 genes encoding uncharacterized hypothetical proteins were annotated as putative virulence factors. These genes were selected because of the presence of predicted features potentially associated with secretion or membrane localization, such as signal peptides (SPs) and transmembrane domains (TMs). The selected loci were named *hp1* to *hp21* in this study (Supplementary Table S1). A subset of these loci overlapped with hypothetical transmembrane protein genes previously investigated by Rossi et al. [16], namely *htmp1* to *htmp5*, corresponding to *hp16*, *hp1*, *hp18*, *hp3*, and *hp7*, respectively.

Based on the CH reference sequence, which belongs to the M54 *map* genotype, new primer pairs were designed for nested-PCR amplification of each candidate locus (Supplementary Table S1). The locus M6G77_00510, corresponding to *hp21* in this study, was excluded from the MLST analysis because of its large size (3180 bp) and the presence of repeated regions along this locus.

Primer pairs for direct and nested PCR were manually designed using BioEdit v7.2.6 [41], preferentially targeting GC-rich regions located near the 5′ and 3′ ends of each gene. For longer genes, approximately >700 bp, primers were designed within the coding sequence to amplify an internal fragment. For shorter genes, approximately <700 bp, primers were designed in the flanking regions outside the open reading frame (ORF) to allow amplification and sequencing of the full-length gene (Supplementary Table S1). Primer properties, including melting temperature and specificity, were evaluated by aligning each primer pair against the complete CH chromosome sequence and by using the web version of Primer-BLAST [42] to minimize non-specific amplification from host plant DNA or other grapevine-associated microorganisms.

### 2.3. PCR Conditions and Sequencing

PCR amplifications were performed using GoTaq DNA Polymerase (Promega Italia, Milan, Italy) in a final volume of 25 μL, according to the manufacturer’s instructions. For both direct and nested PCR reactions, 2 μL of total DNA or first-round PCR product was used as template, respectively. DNA extracted from healthy *Catharanthus roseus* L. plants and no-template reaction mixtures were included as negative controls. Thermal cycling conditions consisted of an initial denaturation at 94 °C for 5 min, followed by 35 cycles of denaturation at 94 °C for 1 min, annealing at 50 °C for direct PCR or 52 °C for nested PCR for 1 min, and extension at 72 °C for 2 min, with a final elongation step at 72 °C for 7 min. For shorter amplicons, the extension time was reduced to 1 min. Nested PCR products were verified by electrophoresis on 1% agarose gels and sequenced in both directions by Eurofins Genomics, Germany.

### 2.4. Sequence and Bioinformatic Analyses

Raw sequences were trimmed and assembled into consensus sequences using BioEdit v7.2.6 [41]. Consensus nucleotide sequences were then translated in silico into the corresponding amino acid sequences. For each locus, nucleotide and amino acid sequence alignments were generated using the ClustalW algorithm [43] implemented in BioEdit v7.2.6, using the corresponding sequence from the FDp reference strain CH as reference [39]. For shorter genes amplified together with flanking regions, only the coding sequence was retained for subsequent analyses. Sequence identity matrices were constructed from nucleotide and amino acid alignments to evaluate sequence identity among FDp genotypes for each locus. Single nucleotide polymorphisms (SNPs) were defined as unique nucleotide positions differing from the CH/M54 reference sequence. For descriptive purposes, locus variability was classified according to the number of SNP positions detected as limited (1–2 SNPs), moderate (3–10 SNPs) or high (>10 SNPs). Gap-containing positions were excluded from SNP counts and considered separately when present. The number and position of SNPs were recorded, and their effects at the amino acid level were determined. Codons containing nucleotide differences but encoding the same amino acid were classified as synonymous, whereas codons encoding different amino acids were classified as non-synonymous. Amino acid substitutions were classified according to the BLOSUM62 substitution matrix [44]: substitutions with a positive BLOSUM62 score were considered conservative, whereas substitutions with a score of zero or lower were considered non-conservative. Sequences generated in this study were deposited in GenBank under accession numbers PZ713514 to PZ713568 (Supplementary Table S2).

Predicted signal peptides and transmembrane domains were re-evaluated using the Phobius web server [45,46], and functional domain annotations were cross-checked using InterProScan [47], in comparison with the reference genome annotation of strain CH [39]. To investigate sequence similarity with previously described phytoplasma secreted proteins, the deduced amino acid sequences of the selected proteins were compared with the secreted AY-WB protein (SAP) repertoire described for ‘*Candidatus* Phytoplasma asteris’ AY-WB [48] using BLASTP v2.16.0+ [49]. In addition, each deduced amino acid sequence obtained in this study for the target loci was used as a query in BLASTP searches against the NCBI non-redundant protein database, restricting the analysis to phytoplasma proteins, to identify related proteins in other phytoplasma species. For these searches, the first 100 hits were inspected, and only alignments showing at least 75% query coverage were considered for comparative evaluation.

Phylogenetic analyses were performed on nucleotide alignments of the most polymorphic loci identified in this study. The amplicon sequence of the *map* region was also analyzed as a reference marker to compare the phylogenetic placement of strains based on the conventional *map*-based classification with that obtained from the candidate effector loci. Multiple sequence alignments were generated in BioEdit using the ClustalW multiple alignment algorithm. Maximum-likelihood trees were inferred in MEGA11 [50] using the Tamura-Nei nucleotide substitution model [51], with uniform rates among sites and all sites included in the analysis. Branch support was assessed using 1000 bootstrap replicates. Unless otherwise specified, default parameters were used for all analyses.

## 3. Results

### 3.1. Gene Selection and Performance of Newly Designed MLST Primers

**Figure 1:**
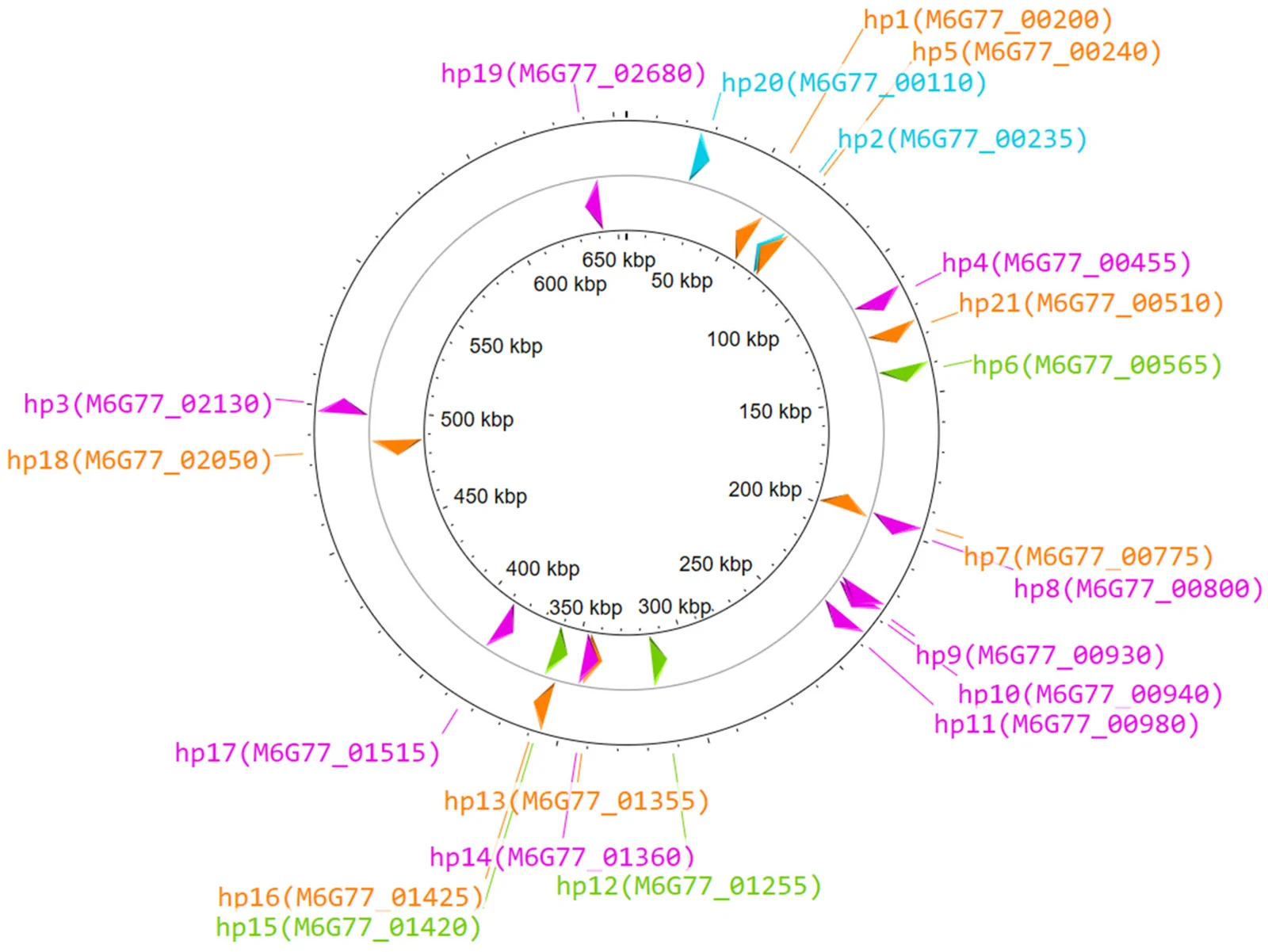
Genomic distribution of the loci selected for MLST analysis in the FDp CH reference genome. Selected loci are indicated by coloured arrows labelled with their corresponding locus tags; arrow direction indicates gene orientation on the chromosome. Colours indicate the predicted protein features: signal peptide (SP; purple), transmembrane domain(s) (TM; orange), both SP and TM domain(s) (blue), or neither SP nor TM domain(s) (green). The outer ring indicates genes encoded on the forward strand, whereas the inner ring indicates genes encoded on the reverse strand. Genome length is indicated in kb on the inner scale. Figure generated with Proksee (build 2026-08-10) [52].

To evaluate the genetic variability among different FDp strains linked to putative effector-coding genes, 21 candidate genes were selected based on the functional annotation of the FDp reference strain CH genome (acc. no. CP097583.1) [39]. The selected loci, named *hp1* to *hp21* in this study, encode hypothetical proteins identified as candidate virulence factors. Their position and orientation within the FDp CH chromosome are shown in Figure 1. 

Predicted protein features were re-evaluated using InterProScan (https://www.ebi.ac.uk/interpro/) and Phobius (https://www.ebi.ac.uk/jdispatcher/) to assess the presence of signal peptides (SP) and transmembrane domains (TM) (Figure 1). Overall, most of the selected proteins were predicted to contain features potentially associated with secretion or membrane localization: seven proteins contained one or two transmembrane domains, nine proteins were predicted to contain a signal peptide only, and two proteins harboured both a signal peptide and a transmembrane domain. Among the proteins carrying only a signal peptide, two also contained an SVM domain, as predicted by InterProScan. All selected loci encoded hypothetical proteins, except for *hp18*, which was the only locus with an assigned predicted function, encoding a Signal Peptidase I according to InterProScan annotation. The predictions obtained in this study were largely consistent with those reported by Debonneville et al. [39], with full or partial agreement observed for 18 out of the 21 selected genes. Differences in domain prediction were observed for a subset of loci–*hp6*, *hp12*, and *hp15*–for which transmembrane domains and/or signal peptides were not confirmed, suggesting a likely cytoplasmic localization. Despite these differences, all loci previously annotated as putative effectors were retained for subsequent analyses, regardless of the predicted domain architecture. 

New primer pairs were designed on the CH reference genome (Supplementary Table S1) to amplify the remaining 20 loci by nested PCR across 36 FDp-infected samples representing different *map* genotypes. Amplification success was highly heterogeneous among loci and genotypes (Table 2). Overall, 16 out of 20 loci were successfully amplified in at least one genotype, whereas four loci (*hp2*, *hp3*, *hp5*, and *hp20*) failed to amplify in all samples. Notably, three of these non-amplified loci (*hp2*, *hp5*, and *hp20*) contained repeated regions, which may have affected primer design or amplification efficiency. The loci *hp13* and *hp14* were detected only in M54 samples, whereas the remaining loci were amplified in multiple genotypes. Among these targets, *hp6*, *hp12*, *hp15*, and *hp18* showed the broadest amplification success across the strain panel and were detected in most genotypes, while *hp1* and *hp17* also showed relatively high amplification frequencies, although with a more restricted genotype distribution. Other loci displayed intermediate or more variable amplification profiles, whereas *hp16* was only sporadically amplified. In most cases, PCR amplification yielded a single band of the expected size, although occasional non-specific products were observed and excluded from subsequent analyses.

From a genotype-centred perspective, M54 samples showed the most consistent amplification profile, with all strains successfully amplified for at least 12 loci and a core set of nine loci detected across all M54 samples, regardless of host plant origin. Among the remaining genotypes, M50 and M51 showed intermediate to high amplification coverage, although with greater variability among strains, whereas M38 and M78 showed the lowest amplification success. Variable amplification profiles were also observed within M39 and M43, indicating that amplification efficiency differed not only among genotypes but also among strains belonging to the same *map* genotype. Complete amplification profiles for each strain and locus are reported in Table 2.

### 3.2. Sequence Variability and SNP Analysis of Candidate Loci

High-quality sequences obtained from positive amplicons (Supplementary Table S2) were aligned with the corresponding loci from the FDp reference genome strain CH, belonging to genotype M54, to assess nucleotide and amino acid variability. SNP analysis revealed different levels of sequence variability among strains belonging to different *map* genotypes (Table 3). No sequence variability was detected within the M54 cluster, as all M54 strains showed complete identity with the CH reference across all successfully amplified loci, regardless of the host plant of origin. In contrast, all other genotypes displayed varying degrees of divergence from the M54 reference. M51 strains differed from M54 in most of the amplified loci, but showed very limited intra-genotype variability, restricted to *hp18*. By contrast, M50 showed the highest level of sequence heterogeneity, with divergence from the CH reference in most amplified loci and distinct SNP patterns identified among strains belonging to the same *map*-type in *hp8*, *hp9*, *hp12*, *hp18*, and *hp19*. The remaining genotypes also differed from M54 at most successfully amplified loci, although the interpretation was partly limited by the lower number of sequences available for some genotypes, particularly M38 and M78. Intra-genotypic variability was also observed for strains belonging to M39 (*hp6* and *hp12*) and M43 (*hp12*, *hp15* and *hp17*). Interestingly, some polymorphisms were shared with other genotypes, while others appeared to be genotype- or strain-specific. Nevertheless, genotype-associated differences generally involved only a limited number of polymorphic positions, indicating that most loci remained highly conserved despite the presence of sequence variants. This was confirmed by sequence identity matrices based on nucleotide and deduced amino acid alignments, which showed pairwise identity values generally above 0.90 at both levels. Lower identity values were restricted to a small subset of loci, mainly *hp8*, *hp18*, and *hp19*. For *hp8* and *hp19*, reduced identity values were associated only with M50 variants, whereas for *hp18* lower identity values were observed among variants belonging to several genotypes. Complete nucleotide and amino acid identity matrices are provided in Supplementary Table S3.

Overall, considering variability on a gene-by-gene basis, sequence differences were unevenly distributed among the target loci. Several genes showed only limited polymorphism, whereas a restricted subset accumulated a higher number of SNPs and amino acid substitutions. Limited variability was observed for *hp6*, *hp7*, *hp9*, *hp11*, and *hp16*, which showed no polymorphism or only one to two SNPs. Moderate variability was detected for *hp4*, *hp10*, *hp15*, *hp17*, and *hp19*, whereas *hp1*, *hp8*, *hp12*, and *hp18* showed the most complex variability patterns. In particular, *hp8* accumulated the highest number of SNPs, mainly due to a highly divergent M50-associated variant, while *hp18* showed both nucleotide substitutions and short indel-associated regions in the translated alignment. The total number of unique SNP positions, synonymous mutations, and conservative or non-conservative amino acid substitutions identified for each locus is reported in Table 3. Extended SNP tables, including nucleotide positions, substitutions, and the corresponding amino acid changes when present, are provided in the Supplementary Table S4 for each variable locus. For the two most variable loci (*hp8* and *hp18*), complete amino acid alignments are reported instead, in order to show the full extent of sequence divergence.

### 3.3. Protein Similarity and Phylogenetic Analysis of the Most Variable Loci

BLASTP analysis of the deduced proteins revealed heterogeneous similarity patterns with proteins from other phytoplasmas. Several Hp proteins showed moderate to high similarity to proteins from related 16SrV phytoplasmas, particularly AldYp and ‘*Ca.* P. rubi’, and more occasionally ‘*Ca*. P. ziziphi’, with identity values ranging from approximately 70% to more than 95%, depending on the locus and variant. This pattern was observed for several deduced proteins, including Hp1, Hp6, Hp9, Hp10, Hp11, Hp18, and Hp19. In contrast, other proteins, such as Hp4, Hp12, Hp13, Hp14, Hp15, and Hp16, retrieved only low similarity matches with other phytoplasma proteins, generally below 50% amino acid identity.

Interestingly, a few loci showed distinct similarity patterns. The highly divergent Hp8 variant detected in M50 strains from *A. altissima* showed higher BLASTP similarity to a hypothetical protein from ‘*Ca*. P. rubi’ (acc.no. WP_268850040.1) than to the FDp M54 reference sequence, with approximately 82% and 69% amino acid identity over 100% query coverage, respectively. By contrast, the remaining Hp8 variants were more closely related to FDp sequences. Hp17 represented another exception, as its best BLASTP hit–excluding FDp–corresponded to a hypothetical protein from ‘*Ca*. P. mali’ (acc.no. WP_012504569.1), with ~94% amino acid identity over 100% query coverage, whereas similarity to proteins from closely related 16SrV phytoplasmas was below 70%.

BLASTP comparison with the secreted AY-WB protein repertoire [48] revealed that only Hp10 and Hp19, both carrying an SVM domain, displayed a broad similarity to SAPs. Hp10 showed the highest similarity to SAP68 with 63.5% amino acid identity over 81% query coverage, and a lower similarity to SAP21 and SAP19, both showing approximately 40% identity over 80% coverage. Hp19 showed similarity to SAP21, with 57.5% amino acid identity on 100% coverage. No close similarity to functionally characterized SAP effectors was detected for the other FDp candidate proteins.

**Figure 2:**
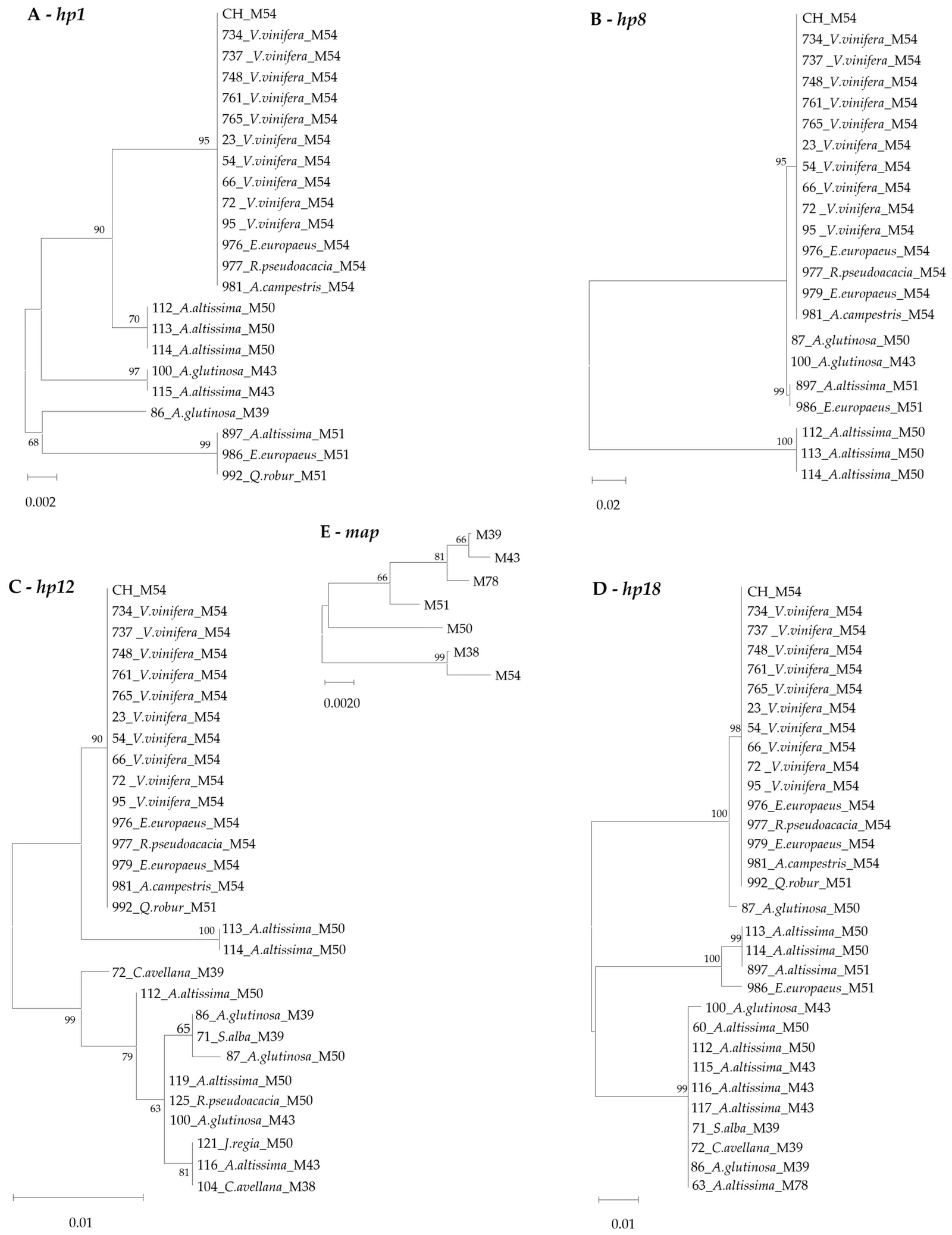
Phylogenetic analysis of the most variable candidate virulence-related loci identified in this study. Maximum-likelihood trees were inferred from nucleotide alignments of *hp1* (**A**), *hp8* (**B**), *hp12* (**C**), and *hp18* (**D**) sequences obtained from the analyzed FDp and FDp-related strains, together with the corresponding reference sequences extracted from the FDp strain CH genome. The *map* tree (**E**) is shown as a reference for the phylogenetic placement of the analyzed strains according to the conventional *map*-based classification. For each strain, labels indicate the strain ID, plant host of origin, and *map* genotype; CH_M54 indicates the reference strain CH. Bootstrap values based on 1000 replicates are shown only when higher than 70. Scale bars indicate nucleotide substitutions per site.

Locus-specific phylogenetic analyses were performed on the most polymorphic loci, namely *hp1*, *hp8*, *hp12*, and *hp18*, and compared with the tree inferred from the *map* amplicon (Figure 2). The *map*-based tree grouped strains according to the expected genotype classification, with M38 clustering close to M54, and the non-epidemic genotypes M39, M43, and M78 forming a separate cluster within the 16SrV-C branch. In contrast, the trees based on candidate virulence-related loci showed locus-dependent clustering patterns. M54 sequences consistently formed a compact group, in agreement with the absence of sequence variability observed for this genotype. By contrast, the position of the other genotypes varied among loci, and strains belonging to the same *map* genotype did not always cluster together. This was particularly evident for M50. In the *hp8* tree, M50 strains were clearly separated into distinct branches: the strain from *A. glutinosa* clustered close to M43, M51, and the M54 group, whereas the divergent M50 variants from *A. altissima* formed a separate branch, consistent with the high sequence divergence observed for this locus. However, the same *A. altissima* M50 strains showed a different placement in other loci, clustering closer to M54 in *hp1* and *hp12*, while the M50 sample from alder clustered close to M54 in *hp18*, but separately in *hp12*. Variable intra-genotype placement was also observed for other genotypes, particularly in *hp12*, where M39 and M43 strains did not form genotype-specific clusters, and in *hp18*, where one M51 from *Q. robur* strain clustered with M54 whereas the remaining M51 strains grouped with M50 variants.

## 4. Discussion

Classical and high-resolution molecular markers have greatly improved FDp strain classification and epidemiological tracking, revealing that genetic diversity may extend beyond *map*-based genotypes [14,16,17]. However, these markers mainly describe population structure and strain circulation, whereas they provide limited information on the variability of genes potentially involved in phytoplasma-host or phytoplasma-vector interactions. In this context, the present study complements conventional FDp genotyping by applying a function-oriented MLST approach to loci annotated as putative effectors or virulence-related proteins in the FDp CH genome [39].

A first important aspect emerging from this study was the heterogeneous amplification success observed among the selected loci and across FDp genotypes. This pattern may reflect, at least in part, true differences in locus conservation among strains, suggesting that some candidate effector genes may be absent, highly divergent, or strain-specific in certain genetic backgrounds. Variation in effector repertoires has also been reported in other phytoplasma groups; for example, in ‘*Ca*. P. asteris’, differences in SAP distribution and abundance between 16SrI-A and 16SrI-B subgroups were proposed to contribute to distinct disease progression dynamics, possibly through effects on insect vector attraction or reproduction [53]. Although this evidence comes from a different phytoplasma pathosystem, it supports the idea that differences in effector-related gene content may have ecological or epidemiological relevance. Nevertheless, because primers were designed on the M54 reference sequence, amplification failure in other genotypes should not be interpreted directly or exclusively as gene absence, as it may also result from sequence divergence at primer-binding sites or other technical constraints, including suboptimal amplification conditions, repeated regions within the target sequence, or differences in DNA quality and PCR inhibitor content among plant samples. This interpretation is consistent with the amplification difficulties reported by Rossi et al. [16] for genes encoding hypothetical transmembrane protein correspondent to some of the loci analyzed here, highlighting that PCR-based analysis of variable genes can be strongly influenced by the selected amplification strategy.

Among the loci for which sequence information was available, the most evident genotype-level result was the complete conservation observed within M54. This conservation was detected across strains from different sampling areas and plant sources, including grapevine and asymptomatic woody hosts, and is consistent with the genetic homogeneity previously reported for this genotype using classical FDp typing markers [13,14,16]. However, M54 should not be regarded as a fully homogeneous population, since fine-scale variability in this cluster has been detected in previous studies using more discriminatory markers [16,17]. Nevertheless, the conservation of the candidate virulence-related loci analyzed here may reflect a stable host-interaction strategy in the M54 strains examined. In contrast to M54, the other analyzed *map* genotypes showed a more complex pattern, characterized by sequence divergence from the CH reference and by variability that was not always consistent with *map*-based classification. For genotypes represented by only a few amplified loci, such as M38 and M78, the limited sequence information reduced the resolution of the comparison. Among the remaining genotypes, M50 strains represented the clearest example of heterogeneity within a single *map*-type, while the others showed a more conserved profile, indicating that the relationship between *map* genotypes and variability at these loci differs among FDp genetic backgrounds. Partial incongruence with conventional FDp strain grouping was further supported by phylogenetic inference based on the most variable genes analyzed here, which generally showed locus-specific strain placements. While M54 isolates remained completely homogeneous, the other strains did not generally form genotype-specific clusters, with some variants shared across different *map* genotypes and others restricted to individual or a few strains. No consistent association with host plant species was evident across loci and, unlike the *map* phylogeny, the separation between epidemic and non-epidemic genotypes was not maintained in any of the trees. This lack of association may indicate that variation in individual candidate genes is not sufficient to explain complex strain phenotypes, which may instead depend on combinations of multiple genes, differences in gene expression, or other genomic and host-related factors. Overall, these findings indicate that candidate virulence-related loci can provide an additional layer of strain differentiation beyond conventional *map*-based classification, although the biological significance of this diversity remains unresolved.

In this context, the different levels of variability observed among loci suggest that the selected candidates may be subject to different degrees of functional constraint or evolutionary divergence. Considering the loci with broader coverage across the analyzed dataset, more conserved genes, such as *hp6*, *hp7*, *hp9*, and *hp11*, may encode proteins whose functions are maintained across FDp strains with different genetic and epidemiological backgrounds. In contrast, the most variable loci identified here, particularly *hp1*, *hp8*, *hp12*, and *hp18*, accumulated non-synonymous and non-conservative amino acid substitutions that could potentially affect protein properties, including structure, localization, activity, or interaction affinity with host or vector components, making them priority candidates for future functional investigation. The potential functional relevance of this variability is supported by studies on other phytoplasma proteins, which have shown that sequence variation can be associated with different biological properties. Among secreted effectors, phyllogens/SAP54-like proteins [54,55] provide one of the clearest examples, as comparative functional analyses showed that a single amino acid polymorphism can affect the ability of these proteins to interact with their target, thereby influencing phyllody-inducing activity [56]. A similar case is represented by SAP11 [57], whose homologues from Aster Yellows Witches’ Broom (AY-WB) and Maize Bushy Stunt (MBSP) phytoplasmas differ in target specificity and produce distinct effects on plant development, defence-related responses, and vector-associated traits, suggesting that sequence divergence among related effectors may contribute to functional diversification [58]. Beyond canonical secreted effectors, sequence variability in membrane-associated proteins has also been linked to differences in biological properties: in FDp, variation in the adhesin-like proteins VmpA and VmpB correlates with transmission by different leafhopper groups, with only specific Vmp clusters associated with *S. titanus* transmission and FD outbreaks [14,59]. However, in this study, the classification of amino acid substitutions provides only a preliminary indication of possible functional effects, and the biological consequences of the polymorphisms observed here cannot be inferred from sequence data alone.

To further contextualize these candidates, BLASTP analyses were conducted to assess their similarity to proteins from other phytoplasmas and to explore whether comparisons with previously described sequences could provide additional information. Overall, the selected proteins showed heterogeneous similarity patterns, and no close similarity to functionally characterized SAP effectors, including SAP05 [60], SAP11 [57], and SAP54 [55], was detected among the FDp candidates analyzed here. This observation is also in line with the comparative genomic analysis of 16SrV phytoplasmas conducted by Böhm et al. [33], which highlighted the presence of SAP11- and SAP54-like proteins in ‘*Ca*. P. rubi’ and ‘*Ca*. P. ziziphi’ but not in the FD-associated CH strain. This pattern is consistent with the absence of typical SAP-associated symptoms in FD-diseased plants, such as phyllody and witches’ broom. Only Hp10 and Hp19 showed detectable similarity to SAPs from the AY-WB phytoplasma repertoire [48], although these matches involved SAPs that have not been functionally characterized, leaving their biological relevance unclear. Beyond the comparison with SAP proteins, the frequent BLASTP similarity observed between several FDp candidates and proteins from related 16SrV phytoplasmas, such as AldYp and ‘*Ca*. P. rubi’, is consistent with their *16S rRNA*-based phylogenetic proximity and suggests that part of the selected gene set may represent a conserved component of the 16SrV protein repertoire. However, this conservation appears to be only partial, as also supported by the comparative genomic analysis of complete 16SrV genomes [33], in which only four predicted secreted proteins were identified as orthologous across all analyzed genomes. In agreement with this, some candidate proteins showed low or no similarity to proteins from other phytoplasmas; however, this should not be interpreted solely as evidence of FDp specificity, but may also reflect rapid sequence divergence and limited conservation, or the still incomplete representation of closely related phytoplasma proteins in available databases. Moreover, the distinct BLASTP patterns observed for the divergent Hp8 M50 variant, which showed higher identity to ‘*Ca*. P. rubi’ than to the CH reference sequence, and for Hp17, which showed higher similarity to ‘*Ca*. P. mali’ than to other 16SrV strains, are particularly noteworthy, as they suggest that some candidate loci may have evolutionary histories that differ from those inferred from the overall FDp strain background. However, comparative genomic analyses including flanking genomic regions would be required to clarify the basis of these similarity patterns and to assess their possible evolutionary significance.

Future studies should extend this analysis to a larger and more diverse collection of strains, including additional *map* genotypes, host species, geographic areas, and epidemiological contexts, and compare variability at putative virulence-related loci with that revealed by high-resolution epidemiological markers reported in previous studies [16,17]. Functional validation and expression profiling will also be required to determine whether the observed sequence variation has biological relevance for host interaction, vector association, or pathogenicity.

In conclusion, this study provides a first function-oriented multilocus analysis of candidate virulence-related genes in FDp and FDp-related strains, revealing a previously underexplored layer of strain-level genetic diversity. Although the biological role of these target genes remains to be validated, the observed variability provides a useful framework for future studies on the molecular basis of FDp diversity and phytoplasma-host/vector interactions.

## Figures and Tables

**Table 1:** FDp-infected samples selected for this study. Samples are grouped according to their *map* genotype and plant host of origin. For each group, the number of analyzed strains, strain IDs, sampling area, and the reference study in which the samples were previously described are reported.

*map* Genotype	Plant Host	No. Strains	Strain IDs	Location	Reference
FDp-M54	*Vitis vinifera*	5	734, 737, 748, 761, 765	Franciacorta	[40]
		5	23, 54, 66, 72, 95	Valtellina	[20]
	*Acer campestre*	1	981	Franciacorta	[40]
	*Euonymus europaeus*	2	976, 979	Franciacorta	[40]
	*Robinia pseudoacacia*	1	977	Franciacorta	[40]
FDp-M51	*Ailanthus altissima*	1	897	Franciacorta	[40]
	*Euonymus europaeus*	1	986	Franciacorta	[40]
	*Quercus robur*	1	992	Franciacorta	[40]
FDp-M50	*Ailanthus altissima*	5	60, 112, 113, 114, 119	Valtellina	[20]
	*Alnus glutinosa*	1	87	Valtellina	[20]
	*Juglans regia*	1	121	Valtellina	[20]
	*Robinia pseudoacacia*	2	123, 125	Valtellina	[20]
FDp-M38	*Corylus avellana*	1	104	Valtellina	[20]
	*Robinia pseudoacacia*	1	106	Valtellina	[20]
FDp-related M39	*Alnus glutinosa*	1	86	Valtellina	[20]
	*Corylus avellana*	1	72	Valtellina	[20]
	*Salix alba*	1	71	Valtellina	[20]
FDp-related M43	*Ailanthus altissima*	3	115, 116, 117	Valtellina	[20]
	*Alnus glutinosa*	1	100	Valtellina	[20]
FDp-related M78	*Ailanthus altissima*	1	63	Valtellina	[20]

**Table 2:** Amplification results of putative virulence-related genes with newly designed primer pairs in different FDp isolates, grouped per *map* genotype. A “+” indicates a positive amplification, whereas empty cells indicate the opposite. Plant species from which FDp isolates were extracted are indicated.

*map* Genotype	Strain ID	Plant Species	Target Locus															
			*hp1*	*hp4*	*hp6*	*hp7*	*hp8*	*hp9*	*hp10*	*hp11*	*hp12*	*hp13*	*hp14*	*hp15*	*hp16*	*hp17*	*hp18*	*hp19*
M54	23	*V. vinifera*	+	+	+	+	+	+		+	+	+	+	+		+	+	
	54	*V. vinifera*	+	+	+	+	+	+	+	+	+	+	+	+		+	+	+
	66	*V. vinifera*	+	+	+	+	+	+	+	+	+	+	+	+	+	+	+	
	72	*V. vinifera*	+	+	+	+	+	+		+	+	+	+	+		+	+	+
	95	*V. vinifera*	+	+	+	+	+	+			+	+	+	+		+	+	
	734	*V. vinifera*	+	+	+	+	+	+		+	+	+	+	+		+	+	
	737	*V. vinifera*	+	+	+	+	+	+		+	+	+	+	+	+	+	+	+
	748	*V. vinifera*	+	+	+	+	+	+	+	+	+	+	+	+		+	+	
	761	*V. vinifera*	+	+	+	+	+	+	+		+	+	+	+		+	+	
	765	*V. vinifera*	+	+	+	+	+	+	+	+	+	+	+	+		+	+	+
	976	*E. europaeus*	+	+	+	+	+	+	+		+		+	+		+	+	
	977	*R. pseudoacacia*	+	+	+		+	+	+	+	+		+	+		+	+	
	979	*E. europaeus*		+	+	+	+	+	+	+	+		+	+		+	+	
	981	*A. campestre*	+	+	+	+	+	+		+	+	+	+	+		+	+	
M51	897	*A. altissima*	+	+	+		+		+					+		+	+	+
	986	*E. europaeus*	+	+	+		+	+						+		+	+	+
	992	*Q. robur*	+	+	+						+			+		+	+	
M50	60	*A. altissima*			+									+			+	
	87	*A. glutinosa*			+	+	+	+		+	+			+		+	+	+
	112	*A. altissima*	+	+	+		+		+		+			+	+		+	+
	113	*A. altissima*	+	+	+		+		+		+			+	+		+	+
	114	*A. altissima*	+	+	+	+	+		+		+			+			+	
	119	*A. altissima*									+							
	121	*J. regia*			+	+					+							
	123	*R. pseudoacacia*			+			+						+				
	125	*R. pseudoacacia*									+							
M38	104	*C. avellana*			+						+							
	106	*R. pseudoacacia*								+						+		
M39	71	*S. alba*									+						+	
	72	*C. avellana*			+						+						+	
	86	*A. glutinosa*	+		+			+	+	+	+			+		+	+	+
M43	100	*A. glutinosa*	+		+	+	+	+		+	+			+		+	+	
	115	*A. altissima*	+		+				+					+			+	+
	116	*A. altissima*			+						+			+			+	
	117	*A. altissima*			+											+	+	
M78	63	*A. altissima*												+			+	

**Table 3:** Summary of nucleotide and amino acid variation detected in each analyzed locus compared with the M54 reference strain CH. The table reports the number of amplified strains and *map* genotypes, the number of distinct nucleotide variants, the total number of polymorphic nucleotide positions, the number of synonymous mutations, and the number of conservative (Conserv.) and non-conservative (Non-conserv.) amino acid (aa) substitutions. Intra-genotype variability indicates the *map* genotype(s) in which more than one nucleotide variant was detected. * Only M54 strains successfully amplified; ** gaps are not included in this count.

Locus	No. Amplified Strains	No. *map* Genotypes	No. Genetic Variants (Nucleotide)	No. SNP Positions	No. Synonymous Mutations	No. aa Substitutions		Intra-Genotype Variability
						Conserv.	Non-Conserv.	
*hp1*	22	5	5	18	6	7	4	no
*hp4*	20	3	2	9	4	4	1	no
*hp6*	31	6	3	2	0	0	2	M39
*hp7*	17	3	2	2	1	1	0	no
*hp8*	21	4	4	102	6	28	34	M50
*hp9*	19	5	2	1	0	0	1	M50
*hp10*	14	5	4	9	1	1	5	no
*hp11*	15	5	2	1	1	0	0	no
*hp12*	28	6	9	16	7	5	3	M50, M43, M39
*hp13*	11	1	1	0 *	0	0	0	No *
*hp14*	14	1	1	0 *	0	0	0	No *
*hp15*	28	6	4	5	1	1	2	M43
*hp16*	4	2	1	0	0	0	0	no
*hp17*	22	6	6	5	2	1	2	M43
*hp18*	30	6	5	54 **	6	13	22	M51, M50
*hp19*	10	5	4	3	0	2	1	M50

## Data Availability

The sequences generated in this study have been deposited in GenBank under the accession numbers from PZ713514 to PZ713568, listed in Supplementary Table S2.

## Supplementary Materials

The following supporting information can be downloaded at https://www.mdpi.com/article/10.3390/pathogens15080850/s1. Table S1: Target loci and primers used for amplification of candidate virulence-related genes; Table S2: Sequence variants and GenBank accession numbers for the amplified candidate virulence-related loci; Table S3: Pairwise nucleotide and amino acid sequence identity matrices for the analyzed candidate virulence-related loci; Table S4: Nucleotide and amino acid variation detected in the analysed candidate virulence-related loci.
